# Quality of life and frailty outcomes following surgical and transcatheter aortic valve replacement

**DOI:** 10.1186/s13019-022-01876-w

**Published:** 2022-05-11

**Authors:** Timothy Luke Surman, John Matthew Abrahams, Jaewon Kim, Hayley Elizabeth Surman, Ross Roberts-Thomson, Joseph Matthew Montarello, James Edwards, Michael Worthington, John Beltrame

**Affiliations:** 1 D’Arcy Sutherland Cardiothoracic Surgical Unit, Royal Adelaide Hospital, North terrace, Adelaide, SA 5000 Australia; 2 Health and Medical Sciences, University of Adelaide, Adelaide, Australia; 3 Cardiology, Queen Elizabeth Hospital, Adelaide, SA Australia; 4 Cardiology, Royal Adelaide Hospital, Adelaide, SA Australia

**Keywords:** Quality of life, Frailty, Depression, Angina, PROMS

## Abstract

**Background:**

Our objective was to report on the prospective outcomes in the areas of depression, quality of life, angina, and frailty in SAVR and TAVR patients with aortic stenosis undergoing aortic valve intervention.

**Methods:**

We recruited 300 patients across 3 groups (TAVR, SAVR, and CABG) over 12 months. Depression, quality of life, frailty, and angina were assessed followed by propensity score matching.

**Results:**

Using logistical regression when all patient factors considered for all patients who had SAVR and TAVR, the only preoperative factors that impacted on 1 year mortality was hypertension and STS score. Quality of life improvements within each group over 12 months was significant (p value = 0.0001). Depression at 12 months between groups (p value = 0.0395) and within each group was significant (p value = 0.0073 for SAVR and 0.0001 for TAVR). Angina was most frequent in TAVR at 12 months in the QL (p = 0.0001), PL (p = 0.0007), and improvement was significant in the QL (SAVR p = 0.0010, TAVR p = 0.0001) and PL (SAVR p = 0.0002), TAVR p = 0.0007) domains in both groups. Frailty at 12 months improved in both groups, but was greatest in TAVR (p value = 0.00126).

**Conclusions:**

This 12 months follow up of cardiac surgical patients has revealed significant improvement in PROMs and frailty in all groups by 3 months postoperative regardless of surgical or transcatheter approach. Outcome measures of quality of life and frailty could be utilized as a measure of outcome more regularly in patients undergoing aortic valve surgery regardless of approach.

## Background

Aortic valve replacement is designed to prolong life and improve its quality, with the latter being particularly relevant given the elderly patient's undergoing this procedure. The early studies reporting on quality-of-life analysis in aortic valve surgery patients were first published in 1997 [1–3]. PROMS were first applied in the areas of heart failure [4] and later to heart valve surgery in 2016 [5]. And determined the value in assessing a patient’s quality of life before and after cardiac surgery.

Our primary endpoint is to determine quality of life between SAVR and TAVR in aortic stenosis (including CABG as a control) over a 12 months period. Our secondary aims are to determine and compare the angina, depression, and frailty outcomes between these groups. We hope that this information will help guide preoperative, perioperative, and postoperative management of patients undergoing aortic valve replacement in these crucial domains that determine patient satisfaction post aortic valve intervention.

## Methods

### Patient recruitment

Following ethics and governance approval (CALHN) (HREC/18/CALHN/188), between June 2018 and August 2020, a total of 300 patients across 3 groups were recruited consecutively from a single institution, at the Royal Adelaide Hospital, Adelaide, South Australia. The 104 three groups comprised a SAVR (100 patients), TAVR (100 patients) and coronary artery bypass grafting (CABG) group (100 patients). All patients were contacted directly, and consent obtained to participate in this data collection that would occur over a 12 months period. Inclusion criteria was patients undergoing a single cardiac procedure (SAVR, TAVR, CABG only) without associated coronary intervention (PCI). Patients excluded had combined procedures, a major perioperative complication precluding continued involvement, patients who died, or who declined involvement. Those patients who declined involvement were replaced with a newly recruited patient to reach the prespecified sample size.

### Baseline demographics

Socio-demographic, symptoms, comorbidities, and risk factors were collected at baseline from the patients as well as hospital records as presented in Table 1.

**Table 1:** Baseline demographics, comorbidities, and cardiac function obtained from the study cohort

Average baseline characteristic	SAVR n = 100	TAVR n = 100	CABG n = 100
Mean age	65.94 (SD 11.6)	82.87 (SD 6.9)	65.90 (SD 10.0)
Gender (male)	79/100	80/100	79/100
Diabetes mellitus	19/100	38/100	46/100
Hypertension	56/100	69/100	74/100
Previous stroke/TIA	5/100	6/100	11/100
AF	10/100	32/100	6/100
eGFR < 90 ml/min	1/100	26/100	8/100
Pulmonary HTN	2/100	2/100	0/100
COPD	13/100	12/100	14/100
Existing PPM	1/100	13/100	0/100
PVD	1/100	10/100	2/100
NYHA class	2.23 (SD 0.7)	2.61 (SD 0.6)	1.13 (SD 0.4)
LVEF	57.95 (SD 8.4)	54.62 (SD 11.8)	54.33 (SD 11.0)
AVA cm^2^	0.99 (SD 0.5)	0.82 (SD 0.3)	2.67 (SD 0.7)
Mean AV gradient	46.57 (SD 15.4)	41.10 (SD 13.8)	5.71 (SD 4.9)
History of CAD	11/100	49/100	100/100
STS score (%)	1.18 (SD 0.4)	4.82 (SD 3.0)	0.77 (SD 0.4)
Cohort mortality	2/100	7/100	0/100

*TIA* transient ischemic attack, *eGFR* estimated glomerular filtration rate, *HTN* hypertension, *COPD* chronic obstructive pulmonary disease, *PVD* peripheral vascular disease, *NYHA* New York Heart Association, *LVEF* left ventricular ejection fraction, *AVA* aortic valve area, *CAD* coronary artery disease, *STS* society of thoracic surgeons

### Health status instruments

Depression was measured using the Patient health questionnaire 9 (PHQ-9) [5–9]. There are 9 domains in the questionnaire with a score assigned 0–3 (0 being no depressive thoughts and 3 being depressive thoughts nearly every day). A range of scores from 0 to 27 are possible. Scores of 5, 10, 15, and 20 represent cut points for mild, moderate, moderately severe, and severe depression, respectively [10].

Quality of life was measured using the Euro QOL EQ-5D questionnaire [5, 11–16]. Quality of life scores were separated into 5 domains with a score of 1–3 giving the patient health profile [17]. A health state score of 1 indicates no problems, a score of 2 indicates some problems, and a score of 3 indicates extreme problems.

Frailty was measured using the Essential Frailty Toolset (EFT) which is a 4-item screening tool incorporating a chair rise activity which is self-reported, any cognitive decline which is reporter assessed, haemoglobin level, and serum albumin level. A score of 3 points indicates frailty [18, 19], while a higher score of > 4 was associated with a reduced 2 years survival [19], and others associated higher all-cause mortality at 1, 2, and 3 years with higher modified EFT scores [20].

Angina was measured using the Seattle Angina Questionnaire (SAQ-7). The SAQ7 consists of 7 questions that reports on activities performed over a 4 weeks period and any specific limitations or symptoms of angina that have impacted on the patient in this time. A score 128 of 0–35 is assigned with 0 indicating the most limitation, pain, and impact on the patient’s quality of life. Three domain scores and one summary score are generated from the SAQ-7 [21].

- A Physical limitation score (SAQ7-PL). The Physical limitation score assesses the degree of physical limitation over the past 4 weeks due to various activities representing mild, moderate, and severe exertion.
- An Angina frequency score (SAQ7-AF). The Angina frequency score assesses the frequency of angina symptoms over the past 4 weeks with higher scores representing lesser angina burden.
- A Quality-of-life score (SAQ7-QL). The Quality-of-life score assesses how the patient perceives their CAD to be impacting his or her QOL.
- A SAQ7 summary score. The SAQ summary score assesses the average of SAQ-PL, SAQ-AF, and SAQ QL scores [21].

### Data collection

Questionnaire data was collected at five independent time periods as inpatient or by telephone questionnaire during the 12 months. The time periods consecutively collected were preoperatively (within 4 weeks of procedure), postoperatively (prior to hospital discharge), 3 months postoperatively, 6 months postoperatively, and 12 months postoperatively.

Data was collected by two investigators over this period, with each investigator reviewing the questioning process and data collection to ensure interobserver reliability. Data analysis was completed by the primary investigator.

### Statistical analysis

Power for recruitment sample size was calculation at 0.05 and 90% power accounting for a 10% dropout rate with 110 patients recruited to satisfy power. Statistical analysis was performed using GraphPad Prism 6 (GraphPad So 152 software, San Diego, California). A p-value of < 0.05 was considered significant.

An unequal variance t-test (Welch’s t test) was used to compare SAVR and TAVR EQ5D health state, and 12 months EQ5D outcomes due to their equal means and normal distribution. A non-parametric test (Mann-Whitney U test) was used to compare EQ5D health score preop and at 12 months in SAVR and in TAVR due to differences in median and not-normally distributed independent groups. It was used to compare SAQ7 preoperative and 12 months scores between SAVR and TAVR, compare preoperative and 12 months scores in the SAVR group and independently in the TAVR group. This was performed in all subdomains of the SAQ7 test. It was used to compare preoperative and 12 months PHQ9 scores between SAVR and TAVR, preop and 12 months scores in the SAVR group and independently in the TAVR group. It was used to compare preoperative and 12 months EFT scores between SAVR and TAVR, and compare preoperative and 12 months scores in the SAVR group and independently in the TAVR group.

Logistical regression followed by propensity score matching was performed using SPSS. We performed a stepwise logistical regression analysis using all known patient preoperative demographics and co-morbidities that were collected. The dependent variable was 1 year mortality. Propensity matching was subsequently performed using the outcome of the logistical regression analysis with a tolerance of up to 1.

## Results

A total of 331 patients were approached during the study to participate in the data collection process. A total of 31 patients declined to be involved for various reasons and subsequently were not included in the data analysis. No patients during the 12 months period declined to continue their involvement in the study, and no patient was lost to follow-up, however 9 patients died through the 12 months data collection period: 7 patients from the TAVR group and 2 patients from the SAVR group.

### EQ-5D depression measurements

**Fig. 1:**
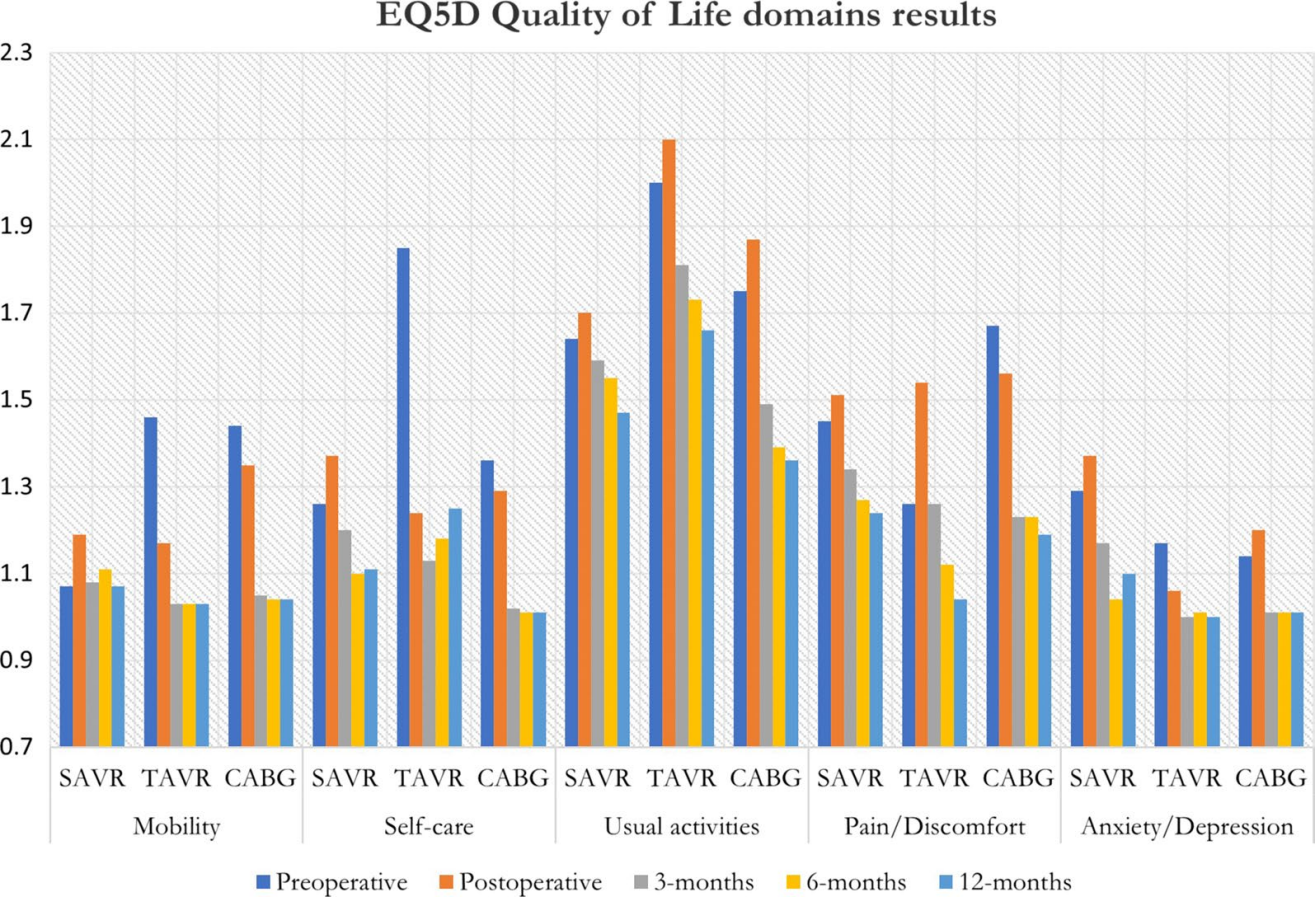
Bar graph showing the distribution of QOL results within each domain amongst all groups

SAVR had the best quality of life regarding mobility (1.10) followed by TAVR and CABG respectively, p = 0.40. In terms of self-care, CABG had the best quality of life (1.01), followed by SAVR and TAVR, p = 0.40. In usual activities, CABG had the best quality of life (1.57) followed closely by SAVR (1.59) and TAVR, p = 0.02 and 0.42 respectively. Pain and discomfort were best in the TAVR group (1.24) followed by SAVR and CABG, p = 0.04 and 0.30. In terms of anxiety and depression symptoms, TAVR reported least symptoms (1.07), followed by CABG and SAVR, p = 0.02 and p = 0.07. The EQ-5D testing domains are summarized in Table 2 and Fig. 1.

**Table 2:** Domain measurements of EQ5D Quality of life in the 3 cohorts over 12-months analysis period

Domains	Mobility			Self-care			Usual activities			Pain/discomfort			Anxiety/depression		
	SAVR	TAVR	CABG	SAVR	TAVR	CABG	SAVR	TAVR	CABG	SAVR	TAVR	CABG	SAVR	TAVR	CABG
Preoperative	1.07	1.46	1.44	1.26	1.85	1.36	1.64	2.00	1.75	1.45	1.26	1.67	1.29	1.17	1.14
Postoperative	1.19	1.17	1.35	1.37	1.24	1.29	1.7	2.10	1.87	1.51	1.54	1.56	1.37	1.06	1.20
3 months	1.08	1.03	1.05	1.2	1.13	1.02	1.59	1.81	1.49	1.34	1.26	1.23	1.17	1.00	1.01
6 months	1.11	1.03	1.04	1.1	1.18	1.01	1.55	1.73	1.39	1.27	1.12	1.23	1.04	1.01	1.01
12 months	1.07	1.03	1.04	1.11	1.25	1.01	1.47	1.66	1.36	1.24	1.04	1.19	1.10	1.00	1.01

Patient’s own perspective of their health status over the 12 months period is summarized in Table 3. The best health status score was in the CABG group at 12 months, followed by TAVR and then SAVR.

**Table 3:** Patient’s own health score given over the 12 months period as a visual analogue scale (VAS) from 0 to 100. A score of 100 indicates the best health a patient perceives themselves to be in at the time

Cohort	SAVR	TAVR	CABG
Preoperative	63.30	57.80	59.50
Postoperative	63.90	70.51	64.00
3 months	72.20	74.44	76.45
6 months	73.50	74.80	79.45
12 months	75.40	75.97	79.65
Average VAS score	69.66	70.70	71.81
Median	72.2	74.4	76.5
IQR	63.6–74.5	64.2–75.4	61.8–79.6

**Fig. 2:**
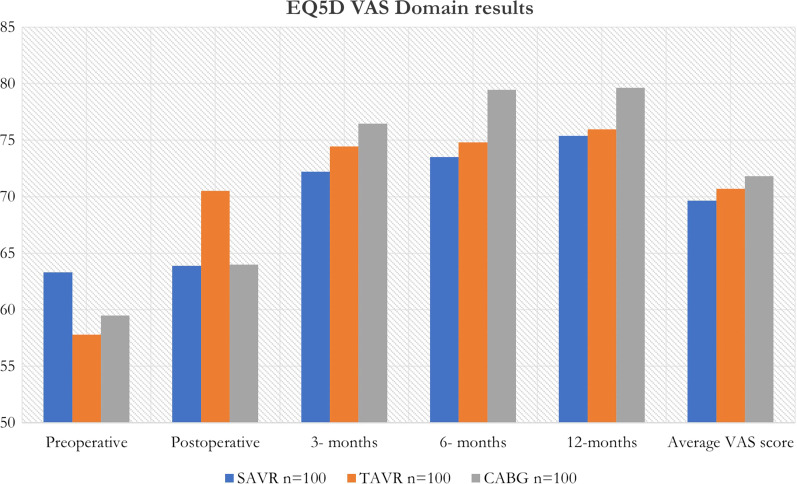
Bar graph showing the distribution of QOL scores according to patients own health score as measured by VAS

Patient results from their own perception of their health or the EQ5D Visual Analogue Scale (VAS) are shown in the Table 3 and Fig. 2.

Each patient’s preoperative and 12 months health state was determined in the SAVR and TAVR groups. Preoperative health state between SAVR and TAVR using an un-paired t-test with Welch’s correction showed a significant difference (p value = 0.02). At 12 months, the SAVR and TAVR groups mean values were the same, and following statistical analysis as above, there was no significant difference between the two (p value = 0.80). When comparing each group separately from preoperative to 12 months health state using the Mann–Whitney U test, SAVR showed a significant difference (p value < 0.0001), and TAVR showed a significant difference (p value < 0.0001).

### PHQ-9 depression measurements

Preoperative depression analysis using Mann–Whitney U test showed significant difference between SAVR (2.31) and TAVR (2.54) (p value = 0.0142). SAVR (median 0.0, IQR 0 – 3); TAVR (median 2, IQR 0–4).

Postoperatively, the range was 0–13 in the CABG group, 0–13 in the TAVR group, and 0–16 in the SAVR group. At 3 months follow-up, depression scores ranged from 0 to 14 in the CABG group, 0-5 in the TAVR group, and 0–16 in the SAVR group. At 6 months follow-up depression scores ranged from 0 to 10 in the CABG group, 0–6 in the TAVR group and 0-15 in the SAVR group. At 12 months, depression scores ranged from 0 to 10 in the CABG group, 0–6 in the TAVR group, and 0–15 in the SAVR group. Postoperative depression analysis using Mann–Whitney U test showed significant difference between SAVR and TAVR (p value = 0.03).

No patients reported symptoms of suicidal or homicidal ideation throughout the questionnaire process. Those who scored higher on the symptom scoring, were referred accordingly. Average depression scores were low in all groups. The SAVR group had the lowest score (1.51) followed by TAVR (1.56) and CABG (1.74) respectively.

Intergroup analysis of preoperative and 12 months depression scores using Mann-Whitney U test showed statistically significant results in the SAVR (p value = 0.01) and TAVR (p value =0.0001).

**Fig. 3:**
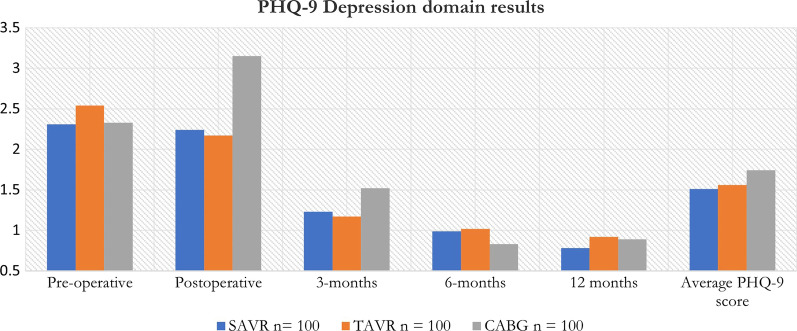
Bar graph showing the distribution of depression scores over 12 months across all groups

Depression measurements as per the PHQ-9 questionnaire over the 12 months data collection period can be summarized in Table 4 and Fig. 3.

**Table 4:** PHQ-9 measure of depression over a 12 months period across the 3 cohorts. A score of < 1 denotes no depressive symptoms and < 5 denotes minimal depressive symptoms

Cohort	SAVR n = 100	TAVR n = 100	CABG n = 100
Pre-operative	2.31	2.54	2.33
Postoperative	2.24	2.17	3.15
3 months	1.23	1.17	1.52
6 months	0.99	1.02	0.83
12 months	0.78	0.92	0.89
Average PHQ-9 score	1.51	1.56	1.74
Median PHQ-9 score	1.23	1.17	1.52
IQR	1.0–2.2	1.0–2.2	0.9–2.3

### EFT frailty measurements

Frailty in the TAVR group was worse preoperatively compared to SAVR. Using the Mann–Whitney U test, this was significantly different (p value = 0.02).

Average frailty scores were higher in the TAVR group (0.98), and CABG group (0.97) compared to the SAVR group (0.83). Noticeably preoperative TAVR frailty scores were higher than the other cohorts (1.08). Only the CABG group in the postoperative measurements (3.15) reached a level of classification as frail. Statistically, the SAVR and TAVR differences at 12 months were not significant (p value = 0.07).

**Fig. 4:**
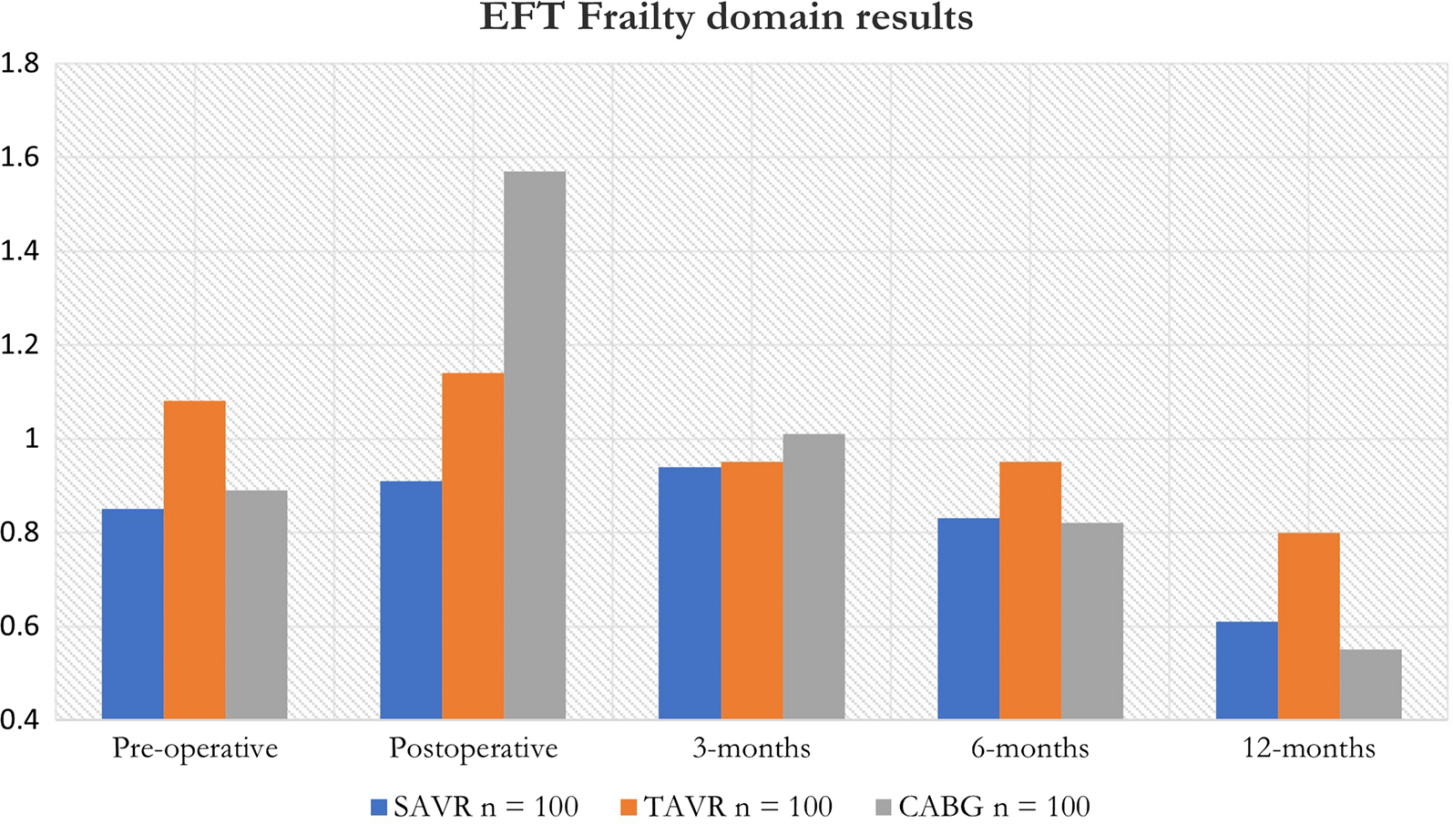
Bar graph showing the distribution of frailty scores over 12 months across all groups

Intergroup analysis revealed no significant difference 225 in frailty over the 12 months in the SAVR group (p226 value = 0.05) and a significant difference in the TAVR group (p value = 0.01). Frailty measurements as per the EFT over the 12 months data collection period are summarized in Table 5 and Fig. 4.

**Table 5:** EFT measurements of frailty over a 12 months period. Scores of 3 or > were classified as frail

Cohort	SAVR n = 100	TAVR n = 100	CABG n = 100
Pre-operative	0.85	1.08	0.89
Postoperative	0.91	1.14	1.57
3 months	0.94	0.95	1.01
6 months	0.83	0.95	0.82
12 months	0.61	0.80	0.55
Average EFT score	0.83	0.98	0.97
Median EFT score	0.85	0.95	0.89
IQR	0.8–0.9	1.0–1.1	0.8–1.0

### SAQ-7 angina measurements

In the measurement of angina outcomes, preoperative scores in the physical limitation (SAQPL) were worse in the CABG group (88.13), followed by TAVR (91.53) and SAVR (94.87) respectively. The difference between SAVR and TAVR preoperatively was significantly different (p value = 0.0002). Scores in the angina frequency (SAQAF) were worse in the CABG group (84.66), and almost equal in the SAVR (99.58) and TAVR (99.91) groups. The difference between SAVR and TAVR preoperatively was not significantly different (p value = 0.1213). Quality of life (SAQQL) was equal preoperatively between CABG (90.50) and TAVR (90.60) and lower in the SAVR group (94.50). The difference between SAVR and TAVR preoperatively was significantly different (p value < 0.0001). Summary scores across all subdomains indicated a higher angina score in the CABG group (87.76), followed by TAVR (94.01) and SAVR (96.32) respectively. The difference between SAVR and TAVR preoperatively was significantly different (p value = 0.0001).

Postoperative scores in the SAQPL group were worse in the TAVR group (91.80), followed by CABG (92.93) and SAVR (94.53). SAQAF scores were higher in the CABG group (90.42), with almost equal scores in the SAVR (99.58) and TAVR (99.91) groups. SAQQL scores were higher in the TAVR group (91.50), with equal scores in the CABG (94.00) and SAVR group (94.10). Postoperative summary score showed higher CABG scores (92.45), followed by TAVR (94.40) and SAVR (96.07).

Scores obtained at 3 months postoperatively in the SAQPL domain showed higher CABG scores (94.80), followed by TAVR (95.47) and SAVR (96.87). Scores in the SAQAF domain showed higher angina scores in the CABG group (95.75) and no reported anginal frequency in both the SAVR (100) and TAVR (100) groups. SAQQL scores were highest in the TAVR group (95.00), followed by almost equal scores in the CABG group (96.10) and SAVR groups (96.20). Summary scores showed higher scores in CABG (95.55) compared to TAVR (96.82) and SAVR (97.90).

Scores obtained at 6 months postoperatively in the SAQPL domain showed higher scores in the TAVR group (96.00), followed by the CABG group (97.33) and SAVR group (98.00). Scores in the SAQAF domain showed higher scores in the CABG group (97.83) with no reported anginal frequency at 6 months in the SAVR (100) and TAVR (100) groups. Scores in the SAQQL domain showed highest scores in the TAVR group (95.30), followed by SAVR (97.6) and CABG (98.30). Summary scores were highest in the TAVR group (97.10) followed by CABG (97.82) and SAVR (98.53).

Scores obtained at 12 months postoperatively in the SAQPL domain showed higher scores in the TAVR group (94.67), followed by CABG (97.33) and SAVR (98.40). The difference between SAVR and TAVR was significantly different (p value = 0.0007). Scores in the SAQAF domain were highest in the CABG group (97.83), followed by TAVR (99.58) and SAVR (100.00). The SAVR and TAVR 12 months scores were significantly different (p value = 0.0251). Scores in the SAQQL domain were highest in the TAVR group (95.80) followed by SAVR (98.10) and CABG (98.30). The 12 months SAQQL scores were significantly different between SAVR and TAVR groups (p value = 0.0001). Summary scores showed higher values in the TAVR group (96.68) followed by CABG (97.82) and SAVR (98.83).

Intergroup analysis showed a significant difference in the preoperative and 12 months SAQPL score in the SAVR group (p value = 0.0002) and TAVR group (p value = 0.0007). Intergroup analysis did not show a significant difference in SAQAF scores in the SAVR group (p value = 0.1213) but was significant in the TAVR group after 12 months (p value = 0.0251). Intergroup analysis showed a significant different in the SAQQL score for SAVR (p value = 0.0010) and TAVR (p value ≤ 0.0001).

**Fig. 5:**
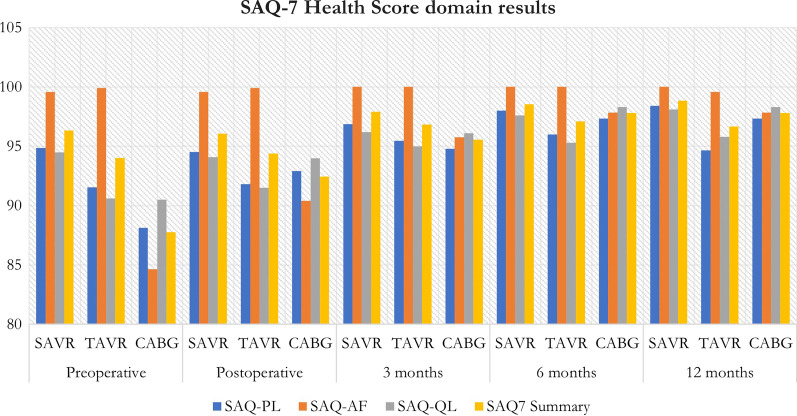
Bar graph showing the domain scores in the SAQ7 questionnaire including the patient SAQ health score over the 12 months study period

Scoring of the subdomains in the SAQ7 questionnaire over the 12 months analysis period can be summarised in Table 6 and Fig. 5

**Table 6:** Summary of the domain scores in the SAQ7 questionnaire including the patient SAQ Health score over the 12 months study period

Score	Preoperative			Postoperative			3 months			6 months			12 months		
	SAVR	TAVR	CABG	SAVR	TAVR	CABG	SAVR	TAVR	CABG	SAVR	TAVR	CABG	SAVR	TAVR	CABG
SAQ-PL	94.87	91.53	88.13	94.53	91.80	92.93	96.87	95.47	94.80	98.00	96.00	97.33	98.40	94.67	97.33
SAQ-AF	99.58	99.91	84.66	99.58	99.91	90.42	100.00	100.00	95.75	100.00	100.00	97.83	100.00	99.58	97.83
SAQ-QL	94.50	90.60	90.50	94.10	91.50	94.00	96.20	95.00	96.10	97.6	95.30	98.30	98.10	95.80	98.30
SAQ7 Summary score	96.32	94.01	87.76	96.07	94.40	92.45	97.90	96.82	95.55	98.53	97.10	97.82	98.83	96.68	97.82

SAVR versus TAVR we matched a total of 58 patients across both groups. Using logistical regression when all patient factors considered for all patients who had SAVR and TAVR, the only preoperative factors that had an impact on 1-year mortality was hypertension, and STS score (Table 7).

**Table 7:** Preoperative variables influencing 1 year mortality outcomes across SAVR and TAVR groups following propensity score matching

Preoperative factor	p value
HTN	p = 0.0368
STS score	p = 0.0040

For the matched patients, we had a higher mean of 34.69 (SAVR) versus 34.07 (TAVR) for SAQ at 1 year which is statistically significant. The remaining results are not statistically significant but because of the low number of matched patients, a determination cannot be made (Table 8). Despite this, clinical significance of these outcomes and comparisons needs to be appreciated.

**Table 8:** Statistical analysis following propensity matching between SAVR and TAVR in all questionnaires

Questionnaire	Mean	p value
SAQ7	SAVR (34.69). TAVR (34.07)	p = 0.0429
PHQ9	SAVR (0.52), TAVR 0.63)	p = 0.6978
EQ5D	SAVR (7.55). TAVR (7.66)	p = 0.9530
EFT	SAVR (0.72), TAVR (0.96)	p = 0.3100

## Discussion

In early registry data, [22] quality of life and frailty was extracted; however, the use of questionnaires was not included, including the PHQ-9, SAQ-7, and EFT. The Partner trials provided randomised outcomes between SAVR and TAVR [22–24], and reported that quality of life and health status were maintained at 12 months [23]. The Partner 2 trial in 2016 assessed baseline heath status using.

Kansas City Cardiomyopathy (KCCQ), SF 36, and EQ 5D questionnaires. This was reported over a 12 years follow-up [24]; and the Partner 3 trial in 2019 assessed functional status and quality of life at 30 days and 1 year using a 6-min walk distance, and (KCCQ) score. Conclusions were that TAVR had rapid improvements in symptoms of failure, 6 min walk distance [22]. Only the Partner 2 trial, used a specific quality of life questionnaire in the use of the EQ5D. In these large trials there has been less focus on quality of life and angina, and no reference towards depression and frailty as primary or secondary endpoints.

This prospective study determined that there is no significant difference in QOL between SAVR and TAVR over a 12 months period. It further went on to explore outcomes in the areas of depression, frailty, and angina in these groups as secondary endpoints.

It should be identified that TAVR patients were much older with more medical comorbidities, compared to the SAVR and CABG control group.

When we summarise the collective findings of the study we find that, quality of life outcomes were evenly distributed across the groups, depressive symptoms improved across all groups, and all groups including the TAVR group improved significantly in the measure of frailty at 12 months. Limited by power calculations and median similarities between median values, frailty results should be interpreted with caution.

Anginal scoring had the most complexity when it came to measurements of outcome. Compared to other instruments, the SAQ was the most responsive instrument to the anginal status and to the clinical change [25]. The SAQ was deemed more responsive than the SF-36 in terms of physical functioning when evaluating patients undergoing coronary bypass surgery (CABG) and angioplasty (PTCA) with a 3 months follow-up after revascularisation [26]. The improvement in physical limitation is noted in both SAVR and TAVR, while anginal improvement was highest in TAVR group compared to SAVR

In comparing the SAVR and TAVR groups, both remained free of significant anginal symptoms throughout the preadmission and postoperative follow-up. The TAVR group started at a higher risk and older age group and despite this had a steady improvement in physical limitations, anginal frequency and quality of life over 12 months.

With the recognised importance in different presentations between men and women in ischemic heart disease [27–29]; measurements in quality of life could also be different in validated instruments. A retrospective multicentre analysis of over 10,000 patients including men and women showed comprehensive evidence that the SAQ is a valid patient-reported instrument that reliably helps capture the symptoms, functional status and quality of life related to angina, while also providing useful prognostic information in women with CAD [17].

In terms of study limitations, this is a prospective cohort study and inherently contains a selection bias, minimised with data collected consecutively. This is supported by a reduced number of propensity matched patients, likely related to lack of power because of reduced patient numbers and therefore reduced statistically relevant conclusions. The EFT has only been validated in the preoperative setting. All PROMS were conducted over the phone and by two investigators, whereas frailty measures were determined through the collection of 345 hospital data and over the phone in the measure of cognitive changes specifically. All three groups had different baselines and comorbidities. The data collection period was only over 12 months and will not capture the intermediate term complications. We recognize that the most important data will occur at least 7 years or more after a procedure when structural valve degeneration can have an impact. In an aim to reduce bias, propensity analysis via logistic regression analysis was performed.

Despite the limitations, the clinical value of such results should not be understated and we hope could supply value to the outcome measures. The SAQ for example is well-established in its validity, reproducibility, prognostic importance, and sensitivity to clinical change, but interpretation can be challenging because of lack of familiarity with the clinical importance of its domains, either cross-sectionally or longitudinally [30]. These questionnaires should be considered tools to support more patient-centred care, and a means of facilitating population health strategies to provide a better foundation for the integration of patient experiences with clinical care.

This study has shown that quality of life, depression, frailty, and angina improves across all groups of varied preoperative risk undergoing interventional and open cardiac surgical procedures over a 12 months period. Clinical evidence supports improvements across all domains and outcome measures for patients who undertake either SAVR or TAVR.

Following aortic valve surgery and coronary bypass surgery, symptoms impacting on a patient’s quality of life reduce by 3 months postoperatively and improve to a point greater than their baseline functioning prior to their surgery regardless of pre-existing age and risk stratification. If we focus on optimising these areas, we may enhance a patient’s perioperative quality of life when undergoing cardiac interventional and open surgical procedures.

## Data Availability

All data incorporated into manuscript.

## Abbreviations

RCT: Randomised control trial
AR: Aortic regurgitation
AF: Atrial fibrillation
KPI: Key performance indicator
PROM: Patient related outcome measure
CABG: Coronary artery bypass grafting
PCI: Percutaneous coronary intervention
eGFR: Estimated glomerular filtration rate
TIA: Transient ischemic attack
PVD: Peripheral vascular disease
COPD: Chronic obstructive pulmonary disease
HTN: Hypertension
AVA: Aortic valve area
STS: Society of thoracic surgery
QOL: Quality of life
EFT: Essential frailty toolset
SAQ: Seattle angina questionnaire
VAS: Visual analogue scale
PTCA: Percutaneous transluminal coronary angioplasty
KCCQ: Kansa City cardiomyopathy questionnaire
ATSI: Aboriginal or Torres Strait Islander
BMI: Body mass index
BSA: Body surface area
EoA: Effective orifice area
PPM: Permanent pacemaker
NYHA: New York Heat Association
LVEF: Left ventricular ejection fraction
MI: Myocardial infarction
CCF: Congestive cardiac failure
IE: Infective endocarditis
CAD: Coronary artery disease
